# Supplementary figures and images for: Dosimetric characteristics of an electron multileaf collimator for modulated electron radiation therapy
Source: J Appl Clin Med Phys. 2010 Apr 12;11(2):5–22. doi: 10.1120/jacmp.v11i2.2913 (PMC5719949; doi:10.1120/jacmp.v11i2.2913)

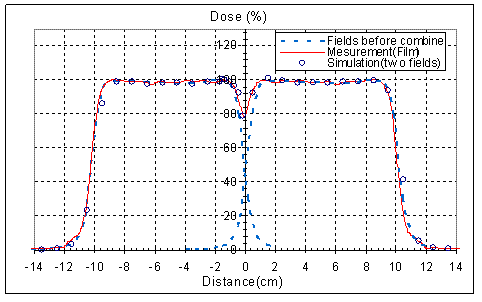

Supplement: Supplementary file 1 — Supplementary Material [file ACM2-11-005-s001.gif]

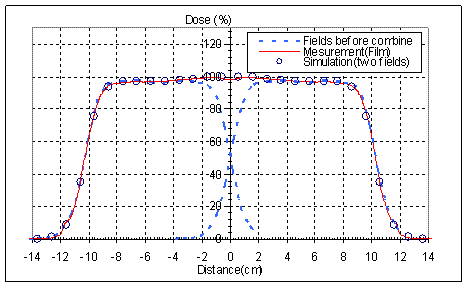

Supplement: Supplementary file 2 — Supplementary Material [file ACM2-11-005-s002.gif]

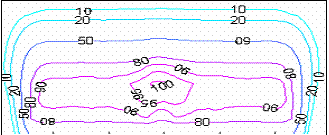

Supplement: Supplementary file 3 — Supplementary Material [file ACM2-11-005-s003.gif]

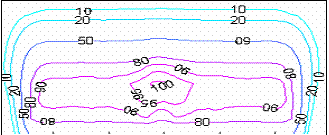

Supplement: Supplementary file 4 — Supplementary Material [file ACM2-11-005-s004.gif]

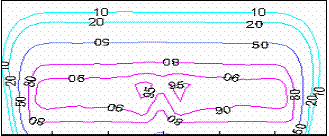

Supplement: Supplementary file 5 — Supplementary Material [file ACM2-11-005-s005.gif]

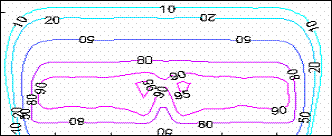

Supplement: Supplementary file 6 — Supplementary Material [file ACM2-11-005-s006.gif]

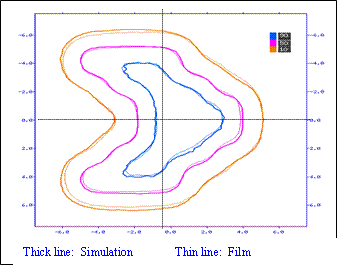

Supplement: Supplementary file 7 — Supplementary Material [file ACM2-11-005-s007.gif]
