# Supplementary material for: Dosimetric characteristics of an electron multileaf collimator for modulated electron radiation therapy
Source: J Appl Clin Med Phys. 2010 Apr 12;11(2):5–22. doi: 10.1120/jacmp.v11i2.2913 (PMC5719949; doi:10.1120/jacmp.v11i2.2913)
Supplement: Supplementary file 8 — Supplementary Material [file ACM2-11-005-s008.html]

Dosimetric characteristics of an electron multileaf collimator for modulated electron radiation therapy


# Dosimetric characteristics of an electron multileaf collimator for modulated electron radiation therapy

> Ahmed A.
> Eldib
> 1a,
> Mohamed I.
> ElGohary
> 1,
> Jiajin
> Fan
> 2,
> Lihui
> Jin
> 2,
> Jinsheng
> Li
> 2,
> C-M Charlie
> Ma
> 2,
> Nader A.
> Elsherbini
> 3  
>  *Department of Physics, Faculty of Science, AlAzhar University, Nasr City, Cairo, Egypt,* 1 *Department of Radiation Oncology, Fox Chase Cancer Center, Philadelphia, PA, USA,* 2 *Department of Oncology, Kasr AlAiny Hospital, Cairo University, Cairo, Egypt*3  
> *ahmedeldeeb2003@yahoo.com
> .*
>
> Received
> 8
> July
> 2009
> : Accepted
> 31
> December
> 2009
>
> Modulated electron radiation therapy (MERT) has been proven as an effective way to deliver conformal dose distributions to shallow tumors while sparing distal critical structures and surrounding normal tissues. It had been shown that a dedicated electron multileaf collimator (eMLC) is necessary to reach the full potential of MERT. In this study, a manually-driven eMLC for MERT was investigated. Percentage depth dose (PDD) curves and profiles at different depths in a water tank were measured using ionization chamber and were also simulated using the Monte Carlo method. Comparisons have been performed between PDD curves and profiles collimated using the eMLC and conventional electron applicators with similar size of opening. Monte Carlo simulations were performed for all electron energies available (6, 9, 12, 15, 18 and 20 MeV) on a Varian 21EX accelerator. Monte Carlo simulation results were compared with measurements which showed good agreement (< 2%/1mm). The simulated dose distributions resulting from multiple static electron fields collimated by the eMLC agreed well with measurements. Further studies were carried out to investigate the properties of abutting electron beams using the eMLC, as it is an essential issue that needs to be addressed for optimizing the MERT outcome. A series of empirical formulas for abutting beams of different energies have been developed for obtaining the optimum gap sizes, which can highly improve the target dose uniformity.
>
> PACS numbers: 87.53.Wz, 87.53.Hv
>
> Key words:
> MERT
> ,
> eMLC
> ,
> abutting electron beams
> ,
> Monte Carlo dose calculation

## I. INTRODUCTION

Some malignant neoplasms are located in close proximity to the patient surface (e.g. head-and-neck tumors (parotid gland), breast and paraspinal lesions) and can be effectively treated by electron beams.(1,2,3) Electron beams have well-defined ranges in water-equivalent tissue and have a sharp dose fall-off beyond the 90% depth dose level (usually considered as the therapeutic dose level(4)). Thus, electron beams offer distinct advantages for superficial and shallow tumors in terms of dose uniformity inside target volume and doses to deeper tissues. Conventional electron treatment use cutouts and bolus to achieve conformal dose coverage.(5) Attempts have always been made to find a practical method for shaping (6,7) and modulating(8,9,10) electron beams that could eliminate the need for cutouts, and thus open the way for modulated electron radiation therapy (MERT) as a new treatment modality. MERT utilizes multiple beams with different energies that are intensity modulated to deliver optimized dose distributions to the target. In the MERT optimization process, lateral dose conformity is achieved by beam shaping while, along the beam direction, the dose conformity is achieved by energy and intensity modulation.(11)

Several studies(12,13,14,15) on modulating the energy and intensity of electron beams have followed the introduction of clinical accelerators based on magnetically scanned pencil beams, and those studies investigated the possibility of using several fluence modulated beams to modify the energy deposition with depth in a controllable manner. This method is not particularly attractive because the lateral spread of the spot beams might be too large at energies below 25 MeV, and the width of the pencil beams in the patient might be too large at energies above 25 MeV.(16)

Another option for electron beams intensity modulation is to use a multileaf collimator. Efforts in this direction can be classified as two categories: investigations to test the feasibility of utilizing the existing photon multileaf collimator (pMLC), and studies that focused on designing an electron specific multileaf collimator. Karlsson et al.(17) and Lee et al.(18) worked on pMLC of Varian machines. They investigated the possibility of redesigning a standard treatment head for electron beam collimation. It was shown that by replacing the air atmosphere inside the treatment head with helium and by reducing the thickness of the monitor chamber, the penumbra width can be significantly reduced and the virtual/effective point source position increased. Another practical solution is to reduce the source-skin distance, while maintaining sufficient clearance for isocentric treatment.(19) Investigations were also done on characterizing electron beams collimated through an existing Siemens pMLC(20) and the possible modification on treatment head for delivery of electron-photon mixed beam radiotherapy.(21,22) It was demonstrated that the penumbra obtained using Siemens pMLC can be significantly sharpened when moving the secondary foil as high as possible in the treatment head with helium atmosphere. The use of pMLC on a Scanditronix Racetrack microtron(23) was also a subject of many studies.(24,25,26,27,28) A race-track microtron with minimized electron scattering and a multileaf collimator for electron collimation will facilitate the isocentric technique as a general treatment technique for electrons.

Other significant efforts were aimed at addressing the feasibility and implementation of specific eMLC mounted on a frame of an existing electron applicator.(29) This alternative approach is more attractive, as a separate eMLC can be manufactured as an add-on device to existing radiotherapy accelerators, whereas the use of the pMLC will likely require a redesign of the complete treatment head and likely not be a simple upgradable solution.(30)

Ma el al.(29) was able to show that their prototype manual-driven eMLC could deliver modulated electron beams for MERT. The technical designs of the electron specific multileaf collimator were explored in several investigations.(18,29,30,31,32,) Tungsten was found to be the best material for the eMLC leaves. Hogstrom et al.(30) studied the dosimetry for a retractable eMLC, where it was designed with the possibility to retract up to 37 cm from the isocenter. Al-Yahya et al.(32) studied the feasibility of using a few leaf electron collimator for shaping any irregular field and demonstrated that highly conformal distribution could be generated.

It is clear that MERT is a promising modality and could be an aid for many patients either to treat or improve their quality of life.(11,33) Thus more research efforts are needed to get this modality into practice. Our group has shown in a previous paper(34) that an in-house Monte Carlo treatment planning system is capable of performing treatment planning and accurate dose calculations for MERT using the pMLC of Siemens Primus accelerator to deliver radiation therapy to the intact breast. However, we believe that a multileaf collimator specific for electrons will be better and more practical for electron beam collimation. We present here the work done on a redesigned prototype eMLC which can be mounted on a Varian accelerator (21EX, Trilogy, iX, etc). Thus our main aim is to first test the feasibility of our newly-designed prototype eMLC for MERT treatment, and then study some of the problems that would face clinical application.

The general characteristics of the prototype eMLC will be studied to demonstrate its adequacy for treatment. In addition, commissioning of the Monte Carlo simulation was performed, as this is an essential step to ensure MERT dose calculation accuracy.

One simple form of the modulated electron therapy is the utilization of multiple abutted electron fields whereby all the fields have a common virtual source position but each of them has its own energy and weight so as to conform the therapeutic dose to the target. Segmented fields can be planned using existing technology – namely, an appropriate 3D treatment planning system. The 3D treatment planning system needs a sufficiently accurate dose calculation and the ability to model beam edges accurately. Unfortunately, manufacturers do not provide a key tool, which could be useful for manually segmenting the field. It should be possible to automate this process by incorporating some basic rules of abutting electron fields into an algorithm.(35)

Many methods have been proposed in the literature to solve the problem of unacceptably large dose variations at the junction of the two adjoining fields.(36,37,38) Most of the methods utilize a beam penumbra modifier, which is not feasible for our eMLC. A simple and more feasible approach is to optimize the gap between the adjacent electron fields.

In this work, investigation is done to find the optimal gap separation that would result in less inhomogeneity problem at the junction area for all of the energies available from our linac. Investigating the optimal gap between adjacent fields can also provide basic information and help achieve the best MERT plan. As the gap size will be confined in the direction perpendicular to leaf motion by the limited size of the leaf width which may not allow for choosing the optimal gap, this study would give us the upper limitations for the future MERT planning.

## II. MATERIALS AND METHODS

### A. The prototype electron MLC

A prototype manually-driven eMLC was used in this study. It consists of 25 tungsten leaf pairs. Each leaf is 2 cm in thickness and 0.6 cm in width. This prototype eMLC was mounted on the treatment head of a Varian 21EX linac. The leaves can slide on the frame to form the desired shape and be maintained in fixed positions by tightening the screws. The eMLC weights around 18 Kg. We also have a design for the future motorized eMLC which weights around 32 Kg. This system will be comparable in size and weight to the Radionics micro MLC system, which has been proven to be practical where it can be attached to the linac gantry without affecting the mechanical accuracy. Results for the present eMLC can be compared and contrasted to earlier eMLCs. It is most appropriate to compare our results with those of Ma et al,(29) as both were designed primarily for MERT. The present eMLC is designed to replace the treatment applicators for a Varian Clinac 2100C, whereas the eMLC by Ma et al. is designed to rest in the 25cm × 25 cm applicator of the same machine. In the present eMLC, tungsten was chosen as the leaf material (whereas Ma et al. used steel). Although our eMLC is ready for MERT treatment delivery, we are not going to use it for treatment as we intend to have a motorized one. PDD curves and profiles for all energies available from the Varian 21EX for a regular 10 cm × 10 cm eMLC field were measured using ionization chamber in a scanning water tank system. Measurement was done at 70 cm SSD and with a gap of 5 cm between the lower surface of the eMLC and the surface of the phantom. A PTW semiflex ionization chamber (model TN31010, PTW, Freiburg, Germany) was used for scanning in water. Depth ionization curve was corrected for the effective point of measurement and converted to percentage depth dose according to the AAPM TG25.(38) Comparisons were done between the PDD curves and profiles from the eMLC and those from a square electron applicator.

### B. The Monte Carlo beam simulation and dose calculation

In this study, we used the MCBEAM(39) and the MCSIM(40) Monte Carlo codes for accelerator simulation and phantom/patient dose calculation, respectively. We carefully simulated the linac with the data provided by Varian. In our model, the incoming electron beam was assumed to be parallel with a Gaussian space distribution and an energy distribution. The simulated linac head includes scattering foil, ion chambers, upper and lower jaws, exiting window and the eMLC. The energy cutoffs for electron transport in the accelerator simulation (ECUT and AE) were 700 keV (kinetic + rest mass), and for photon transport (PCUT and AP) 10 keV. The electron transport step length was confined such that the maximum fractional energy loss per electron step is 4% (ESTEPE = 0.04). The electron beam emerging from the exit window is usually not monoenergetic and is represented by a spectrum of energies which is not precisely supplied by the manufacturers. Thus for each of the energies, we first simulated with several monoenergetic electron beams. Then an optimization code using the random-creep method was used to derive the energy spectrum for the electron beam to match the measured percentage depth doses (PDD). The derived spectrum was used in the MCBEAM simulation for generating the phase space file. The phase space files were generated at a plane right above the eMLC. All the energies available for the 21EX machine (6, 9, 12, 16, and 20 MeV) were simulated. The MCSIM code was used to simulate the dose distributions in a water phantom for beams collimated by the eMLC using the generated phase space files. Monte Carlo is benchmarked using measurement for 10 cm × 10 cm applicator and measurement done with eMLC in place, where regular and irregular shapes were formed by positioning leaves of the eMLC. Selected shapes were then simulated including simple square field and some shaped beams. Monte Carlo simulated dose distributions were compared with these measurements. Matching between actual machine output and simulation was done by a factor that was calculated and incorporated in MCSIM code for each of the electron beam energies.

### C. Abutment of adjoined fields

Simulations were done with different beam energies for two adjoined eMLC fields to test the performance of our code in inhomogeneous dose situations. Simulations were validated by comparing to film measurements. Films were calibrated under the AAPM TG 25(38) recommendations and were scanned by a spatial resolution of 0.0356 cm.

One way to solve the problem of abutting fields is to optimize the gap separation between the adjacent fields. Investigation was also done to find the optimal gap that should be used for our eMLC when adjoining electron fields (eMLC segments). Gaps were determined by achieving a uniform dose for two adjoined eMLC fields at a desired depth. Dose distributions and dose volume histograms (DVH) were studied to find out the gap that could give less inhomgeneity problem concerning cold and hot spots.

## III. RESULTS & DISCUSSION

### A. PDDs and profiles for an eMLC (measurement)

Figure 1 shows the measured PDDs for all available energies for 10 cm × 10 cm field using applicator compared to one of the same size formed by the eMLC. The PDD for beams shaped with the eMLC shows similar results in the buildup region while slight differences after Dmax to those from the applicator for all energies. Figure 2 shows the profiles taken at two different depths (Dmax and R90 depths) for the applicator and the eMLC. Profiles from the eMLC show similar penumbra to that from the applicator for all energies. For the 6 and 9 MeV beams, profiles from the eMLC have better flatness compared to that from the applicator. The reason for the different flatness at lower energies may be due to the increased in-scatter effect. This effect decreases at higher energies. Penumbra is also calculated for different field sizes and is plotted as a function of field size in Fig. 3. Symmetry and flatness of eMLC collimated beams are calculated for all available energies and are tabulated in Table 1. The virtual point source location for applicator cutout collimation system was calculated to be approximately 90.2 cm away from the isocenter, while it’s approximately 64 cm when the eMLC is attached and the phantom placed at 70 cm SSD. This calculation was needed for matching between the areas of fields shaped by cutouts and those shaped by eMLC for the previous comparison. The effective SSD was also calculated and was found to be equal to approximately 64 cm for the eMLC setup while, for the case of the electron applicator, it was approximately 87 cm.

|  |  |  |
| **Fig. 1**.  PDD curves for applicator and eMLC of 10 cm × 10 cm field for 6 MeV, 9 MeV, 12 MeV, 16 MeV and 20 MeV beams. | | |

|  |  |  |
| **Fig. 2**. Measured profiles at two different depths (Dmax and R90) for applicator and eMLC of 10 cm × 10 cm field for 6 MeV beam. | | |

|  |  |  |
| **Fig. 3**.  Penumbra plotted as a function of energy at different field size (side of square field in cm) formed by eMLC. | | |

**Table 1**. 
Flatness and symmetry of eMLC collimated beams for all available energies.

### B. Characteristics of electron beams collimated by an eMLC

Figure 4 shows the characteristics of 6 MeV and 16 MeV electron beam collimated by the eMLC for same given monitor units to test that the leaf shape is distinguishable and is not blurred due to electron scattering. In addition, this test evaluates the dose reduction when the leaf pairs alternated between open and closed. Film measurements were performed to get profiles in the direction shown in Fig. 4(a). It can be seen in Fig. 4(b) that, even when blocked by only one leaf, a dose reduction to 52% is achieved for 6 MeV and to 35% for 16 MeV. Figure 4(c) shows the effect of using different beamlet widths; it is clear that the designed leaf width for this eMLC is appropriate. One of the concerns regarding the application of eMLC in MERT was the X-ray component that accompanies all electron beams. Figure 5 shows the X-ray component as a function of energy. The X-ray component at 20 MeV electron beam energy is relatively high to the extent that may limit the use of the 20 MeV beam for modulated electron radiation therapy. Another concern was leakage. However, as is shown in Fig. 6, even with the highest energy used the maximum leakage was 3%, while for 16 MeV it was even less than 2%. Figure 7 shows the ratio of output measured for the eMLC to those obtained with a 10 cm× 10 cm applicator electron beam for various electron beam energies. The output measured at Dmax depth was higher for beams shaped with the eMLC than those for applicator of the same size by 50%. This is mainly due to inverse square law, as the measurement was done at 70 SSD in the eMLC case. However, no significant pattern was observed in the variation of the ratio of the output of the eMLC to that of applicator. The presented MERT system is used at 70 cm SSD. However, it is planned that the future motorized eMLC will be at lower position than the current prototype and, as a result, larger SSD will be used. In the present work we only try to solve the problems related with the application of the eMLC.

|  |  |  |
| **Fig. 4**.  Profiles for 6 MeV and 16 MeV (a): the field pattern collimated by the eMLC and the location of profile measurement; (b) profiles at A; (c) profiles at B. | | |

|  |  |  |
| **Fig. 5**.  X-ray component versus energy of the electron beam for eMLC. | | |

|  |  |  |
| **Fig. 6**.  Ratio of leakage to 10 cm × 10 cm open field versus energy. | | |

|  |  |  |
| **Fig. 7**.  Output factor plotted versus field size (side of square field in cm). | | |

### C. The 10 cm × 10 cm applicator simulation

The purpose of verification of the beam model is to ensure that parameters, such as the incident beam energy (if used), are correctly “tuned” to produce dose distributions in agreement with measurement as stated in task group 105.(41) Calculated PDD curves for a 10 cm × 10 cm electron applicator are compared to ionization chamber measurements for energies 6, 9, 12, 16 and 20 MeV, as plotted in Fig. 8. Measured profiles taken at two different depths, Dmax and R90, for all the energies are compared to simulations.

|  |  |  |
| **Fig. 8**.  PDD curves from simulation and measurement for 10 cm × 10 cm field using applicator. | | |

Figure 9 shows profiles at two different depths for 12 MeV beam. Good agreement was achieved between Monte Carlo simulated PDD curves and profiles with measurements.

|  |  |  |
| **Fig. 9**.  Measured profiles compared to simulation at two different depths (dmax and R90) for applicator 10 cm × 10 cm field for 12MeV beam. | | |

### D. The eMLC simulation

Figure 10 shows calculated and measured PDD curves for the 10 cm × 10 cm field collimated by the eMLC for 6, 9, 12, 16, and 20 MeV electron beams. Figure 11 shows comparisons of simulated and measured profiles for the same eMLC field. Good agreement (2%/1mm) was achieved between Monte Carlo simulations and measurements for PDD curves collimated by both electron applicators/cutouts and by the eMLC for all electron energies. The Monte Carlo dose distributions resulting from electron fields collimated by the eMLC agree with ion chamber and film measurements. Figure 12(a) shows a comparison of the calculated isodose distribution for an eMLC shaped field for a 12 MeV beam and experimental measurement done by film. Two cross profiles were taken to further verify the agreement between the simulation results and measurements. Figures 12(b) and (c) show the cross profiles at both vertical and horizontal directions.

|  |  |  |
| **Fig. 10**.  PDD curves simulation and measurement for all the energies for opened 10 cm × 10 cm eMLC at 70 cm SSD. | | |

|  |  |  |
| **Fig. 11**.  Profiles from simulation and measurement at two different depths for eMLC 10 cm × 10 cm defined at 70 cm SSD for 16 MeV beam. | | |

|  |  |  |
| **Fig. 12**.  Irregularly shaped beam (a): isodose distribution of a pattern shaped by eMLC at 12 MeV beam obtained by simulation and film; (b) cross profile in the vertical direction; (c) cross profile in horizontal direction. | | |

### E. Adjoining of two fields

The ability of our code to detect the inhomogeneity arising from the adjoining of two electron beams in the junction area was tested by comparing the simulation results with film measurements for two adjacent beams. Figure 13 shows two adjacent 10 cm × 10 cm fields of 12 MeV beam energy showing a hot spot situation. Film measurements validated the simulated results and demonstrated the ability of our code to perform accurate dose calculation. Based on the Monte Carlo results, we could further optimize the field separation to minimize the inhomogeneity effect in the junction area.

|  |  |  |
| **Fig. 13**.  Simulated and measured profile of two adjacent fields collimated by eMLC with 12 MeV beams: (a) profile before optimizing the gap (gap = 0.3 cm); (b) profile after choosing an optimized gap (gap = 0.6cm). | | |

### F. The optimal field gap

Dose distributions inside a phantom for two adjoined electron fields were investigated for different gaps and different electron energies. As the electron beam from a linac is always divergent, in the adjoined field there must be a point at a specific depth where beyond this point the area tends to be hot and, prior to this point, tends to be cold, depending on the gap size between the fields. Increasing the gap size will further increase the cold area while decrease the hot area, and vice versa. Thus by choosing an optimal gap we can compromise this effect. Dose distribution and dose volume histogram for abutting beams were evaluated; the optimal gap is the one that would result in less inhomogeneity within the specified target area which is covered by the 90% dose line. Gaps were then evaluated by achieving the best dose distributions and the sharpest target DVH curve fall-off. Figure 14 shows dose distribution for abutting fields for 6 MeV beams. Figure 15 shows the DVH for different gap sizes between adjoined electron beams for a specified target in the phantom. Table 2 shows the gap sizes that gave the best dose distributions and DVHs for all the energies available of the Varian 21EX linac at 70 cm SSD. If these gaps are used, a uniform profile can be obtained at Dmax of each corresponding energy.

|  |  |  |
| **Fig. 14**.  Dose distribution for two abutting 6 MeV beams with different separation gap sizes: (a) 0.3 cm; (b) 0.4 cm; (c)0.5 cm. | | |

|  |  |  |
| **Fig. 15**.  DVHs for two adjacent fields on a specified target with different gap sizes between the fields: (a) 6 MeV; (b)9 MeV. | | |

**Table 2**. Optimal gap separation between adjacent electron beams for different energies at 70 cm SSD that would result in uniform profile at Dmax for each of the energies.

Figure 16 shows the gap size required to get a flat profile at depth equal to half the range of the electron beam as a function of energy. It is shown that as energy increases, the gap size needs to be increased (which may be ascribed to the change in penumbra). As we already know, the penumbra of the electron beam increases with energy; therefore, a larger gap size is required to get the required matching.

|  |  |  |
| **Fig. 16**.  Gap size that results in flat profile at depth position equal to half the range of the electron beam as a function of the beam energy. | | |

In clinical practice, source to skin distances are different for different cases. As penumbra also increases with increasing SSD, this means that the gap size should also increase with an extended SSD. Figure 17 shows profiles taken at Dmax for 6 MeV electron beam at 70 cm and 75 cm SSD. Gap size as a function of energy and SSD for gaps chosen to get a flat profile at depth of 3 cm in the phantom are shown in Fig. 18. As we expected, the optimal gap is larger with larger SSD and higher energies. For each certain energy, the gap size shows a linear relationship with the SSD. As an example, for 9 MeV, 12 MeV and 16 MeV beams, gap sizes in the case of 70 cm SSD can be calculated by the following equations, respectively:

|  |  |  |
| **Fig. 17**. Measured profiles at Dmax for 6 MeV electron beam at 70 and 75 SSD. | | |

|  |  |  |
| **Fig. 18**.  The gap size resulting in flat profile at a specified depth as a function of SSD and beam energy. | | |

|  |  |
| --- | --- |
|  |  |

It should be mentioned that this is just a simple example for solving the problem of abutting electron fields that could be useful in the simplest form of MERT by manually segmenting the fields utilizing the existing planning systems. Similar linear equations can be derived for other energies at different depths and with different SSDs, and can be incorporated into the planning system to compromise the problem of inhomgeneity for segmented field treatment. In the previous example, we did not include the complicated situation as it can be handled in our in-house Monte Carlo-based treatment planning system under development.

As shown in Table 2, the smallest value tabulated for the optimal gap is 0.4 and the highest is 0.7 cm; in between these two ranges, the gap size varies depending on the energy combination. This would suggest that the eMLC leaf width should allow for this variation in order to resolve and compromise for the inhomogeneity arising from abutting of field in MERT plane, and would aid the optimization process. In the Lee et al. study,(33) the authors pointed out that, in order to provide the maximum utility at both high and low energies, a 5 mm leaf width was the ideal leaf width for the eMLC. Based on their findings, they concluded that a leaf width of less than 10 mm at 100 cm SSD is not necessary for defining the shape of a low energy field, and leaves of less than 5 mm are not necessary for defining the field shape of any energy. However, based on the results in this study, leaves less than 5 mm width are useful in resolving the variation of the required gap size with different energies. Thus it may be preferable to have leaves of smaller width but limiting their use only for setting gaps between the abutting fields.

## IV. CONCLUSIONS

The prototype eMLC mounted on the treatment head can provide adequate beam collimation for the MERT. There is no significant change in PDDs, and profiles for different electron beams collimated with the eMLC compared to that from the normally used electron applicator at 100 cm SSD. The good agreement between the measured PDDs from a square field shaped by the eMLC and an electron applicator in the buildup region illustrated that the eMLC will not lead to higher surface dose.

We have shown that Monte Carlo simulations are capable of accurately modeling the electron beam delivered by the eMLC. For each electron beam energy, an optimum gap can be chosen to minimize the dose inhomogeneity arising from adjoining two electron fields, which will facilitate the design of the leaf sequence for MERT beam delivery using an eMLC.

## REFERENCES

1. Tapley ND, editor. Clinical Applications of the Electron Beam. New York: Wiley; 1976.

2. Holt JG, Perry DJ. Some physical considerations in whole skin electron beam therapy. Med Phys. 1982;9(5):769–76.

3. Nair RP, Nair TKM, Wrede DE. Shaped field electron dosimetry for a Philips SL 75/10 linear accelerator. Med Phys. 1983;10(3):356–60.

4. International Committee on Radiation Units and Measurements. Radiation dosimetry: electron beams with energies between 1 and 50 MeV. ICRU Report 35. Bethesda, MD: IRCU; 1984.

5. Asell M, Hyödynmaa S, Gustafsson A, Brahme A. Optimization of 3D conformal electron beam therapy in inhomogeneous media by concomitant fluence and energy modulation. Phys Med Biol. 1997;42(11):2083–2100.

6. Bjarngard B, Pionteck R, Svensson G. Electron scattering and collimation system for 12-MeV linear accelerator. Med Phys. 1976;3(3):153–58.

7. Bukovitz A, Saini D, Gur D, Sweeney L. Dosimetric evaluation of a variable field electron beam applicator. Med Phys. 1980;7(3):226–29.

8. Leavitt DD, Stewart JR, Moeller JH, Earley L. Optimization of electron arc therapy doses by multi-vane collimator control. Int J Radiat Oncol Biol Phys. 1989;16(2):489–96.

9. Leavitt DD, Stewart JR, Moeller JH, Lee WL, Takach GA. Electron arc therapy: design, implementation and evaluation of a dynamic multi-vane collimator system. Int J Radiat Oncol Biol Phys. 1989;17(5):1089–94.

10. Leavitt DD, Stewart JR, Earley L. Improved dose homogeneity in electron arc therapy achieved by multiple-energy technique. Int J Radiat Oncol Biol Phys. 1990;19(1):159–65.

11. Ma CM, Ding M, Li JS, Lee MC, Pawlicki T, Deng J. A comparative dosimetric study on tangential photon beams, intensity-modulated radiation therapy (IMRT) and modulated electron radiotherapy (MERT) for breast cancer treatment. Phys Med Biol 2003;48(7):909–24.

12. Karlsson M, Nysttrön H, Svensson H. Electron beam characteristics of the 50-MeV racetrack microtron. Med Phys. 1992;19(2):307–15.

13. Lief E, Larsson A, Humm J. Electron dose profile shaping by modulation of a scanning elementary beam. Med Phys. 1996;23(1):33–44.

14. Ebert M, Hoban P. Possibilities for tailoring dose distributions through the manipulation of electron beam characteristics. Phys Med Biol. 1997;42(1):2065–81.

15. Hyödynmaa S, Gustafsson A, Brahme A. Optimization of conformal electron beam therapy using energy- and fluence-modulated beams. Med Phys. 1996;23(5):659–66.

16. Kudchadker RJ, Hogstrom KR, Garden AS, McNeese MD, Boyd RA, Antolak JA. Electron conformal radiotherapy using bolus and intensity modulation. Int J Radiat Oncol Biol Phys. 2002;53(4):1023–37.

17. Karlsson MG, Karlsson M, Ma CM. Treatment head design for multileaf collimated high-energy electrons. Med Phys. 1999;26(10):2161–67.

18. Lee MC, Jiang SB, Ma CM. Monte Carlo and experimental investigations of multileaf collimated electron beams for modulated electron radiation therapy. Med Phys. 2000;27(12):2708–18.

19. Klein EE, Li A, Low DA. Feasibility study of multileaf collimated electrons with a scattering foil based accelerator. Radiother Oncol. 1996;41(2):189–96.

20. du Plessis FC, Leal A, Stathakis S, Xiong W, Ma CM. Characterization of megavoltage electron beams delivered through a photon multi-leaf collimator (pMLC). Phys Med Biol. 2006;51(8):2113–29.

21. Faddegon BA, Svatos M, Karlsson M, Karlsson M, Olofsson L, Antolak JA. Treatment head design for mixed beam therapy [Abstract]. Med Phys. 2002;29(6):1285.

22. Cheng C, Das I, Srivastava S, Both S. Feasibility study of focused and non-focused photon MLC for electron IMRT (Abstract]. Med Phys. 2006;33(6):2104.

23. Brahme A. Design principles and clinical possibilities with a new generation of radiation therapy equipment. A review. Acta Oncol. 1987;26(6):403–12.

24. Moran JM, Martel MK, Bruinvis IA, Fraass BA. Characteristics of scattered electron beams shaped with a multileaf collimator. Med Phys. 1997;24(9):1491–98.

25. Karlsson MG. and Karlsson M. Electron beam collimation with focused and curved leaf end MLCs – experimental verification of Monte Carlo optimized designs. Med Phys. 2002;29(4):631–37.

26. Zackrisson B, Karlsson M. Matching of electron beams for conformal therapy of target volumes at moderate depths. Radiother Oncol. 1996;39(3):261–70.

27. Essers M, Eggen M, Binnekamp D, Creutzberg CL, Heijmen BJ. Chest wall irradiation with MLC-shaped photon and electron fields. Int j Radiat Oncol Biol Phys. 2000;48(4):1205–17.

28. Karlsson MG, Zackrisson B. Matching of electron and photon beams with a multi-leaf collimator. Radiother Oncol. 1993;29(3):317–26.

29. Ma CM, Pawlicki T, Lee MC, et al. Energy- and intensity-modulated electron beams for radiotherapy. Phys Med Biol. 2000;45(8):2293–311.

30. Hogstrom KR, Boyd RA, Antolak JA, Svatos MM, Faddegon BA, Rosenman JG. Dosimetry of a prototype retractable eMLC for fixed-beam electron therapy. Med Phys. 2004;31(3):443–62.

31. Ravindran BP, Singh IR. R., Brindha S and Sathyan S. Manual multi-leaf collimator for electron beam shaping—a feasibility study. Phys Med Biol. 2002;47(24):4389–96.

32. Al-Yahya K, Hristov D, Verhaegen F, Seuntjens J. Monte Carlo based modulated electron beam treatment planning using a few-leaf electron collimator – feasibility study. Phys Med Biol. 2005;50(5):847–57.

33. Lee MC, Deng J, Li J, Jiang SB, Ma CM. Monte Carlo based treatment planning for modulated electron beam radiation therapy. Phys Med Biol. 2001;46(8):2177–99.

34. Jin L, Ma C-M, Fan J, Eldib A, et al. Dosimetric verification of modulated electron radiotherapy delivered using a photon multileaf collimator for intact breasts. Phys Med Biol. 2008;53(21):6009–25.

35. Hogstrom KR, Antolak JA, Kudchadker RJ, Ma C-M, Leavitt DD. Modulated electron therapy. In: Intensity Modulated Radiation Therapy. The State of the Art: Proceedings of the 2003 AAPM Summer School. Madison, WI: Medical Physics Publishing; 2003. p.749–786.

36. Kurup R, Wang S, Glasgow G. Field matching of electron beams using plastic wedge penumbra generators. Phys Med Biol. 1992;37(1):145–53.

37. Lachance B, Tremblay D, Pouliot J. A new penumbra generator for electron fields matching. Med Phys. 1997;24(4):485–95.

38. Khan FM, Doppke KP, Hogstrom KR, et al. Clinical electron beam dosimetry; report of AAPM Radiation Therapy Committee Task Group No. 25. Med Phys. 1991;18(1):73–109.

39. Li JS, Yang J, Qin L, Xiong W, Ma CM. MCBEAM – A Monte Carlo Tool for Beam Simulation. In: Proceedings of the 14th International Conference on the Use of Computer in Radiation Therapy (ICCR). Seoul, South Korea: Jeong Publishing; 2004. p.541–44.

40. Ma C-M, Li J, Pawlicki T, et al. MCSIM: a Monte Carlo dose verification tool for radiation therapy treatment planning and beam delivery [Abstract]. Med Phys. 2002;29(6):1316.

41. Chetty LI, Curran B, Cygler JE, et al. Report of the AAPM Task Group No. 105: Issues associated with clinical implementation of Monte Carlo-based photon and electron external beam treatment planning. Med Phys 2007;34(12)4818–53.

---


---

a Corresponding author: Ahmed A. Eldib, Department of Physics, Faculty of Science, AlAzhar University, 11884, Nasr City, Cairo, Egypt; phone: 202 011 435 7334; fax: 202 2262 9356; email: 
ahmedeldeeb2003@yahoo.com

(Return to Top)

---


---

Journal of Applied Clinical Medical Physics
, Vol.
11
, No.
2
,
Spring
2010
